# Disease externalities and net nutrition: Evidence from changes in sanitation and child height in Cambodia, 2005–2010

**DOI:** 10.1016/j.ehb.2016.10.002

**Published:** 2016-12

**Authors:** Sangita Vyas, Phyrum Kov, Susanna Smets, Dean Spears

**Affiliations:** a Research Institute for Compassionate Economics; b World Bank Water and Sanitation Program East Asia and the Pacific, 113 Norodom Blvd, Phnom Penh, Cambodia; c Economics and Planning Unit, Indian Statistical Institute, New Delhi, India; d University of Texas, Austin, United States

**Keywords:** Height, Early-life health, Disease environment, Sanitation, Cambodia

## Abstract

Child height is an important indicator of human capital and human development, in large part because early life health and net nutrition shape both child height and adult economic productivity and health. Between 2005 and 2010, the average height of children under 5 in Cambodia significantly increased. What contributed to this improvement? Recent evidence suggests that exposure to poor sanitation – and specifically to widespread open defecation – can pose a critical threat to child growth. We closely analyze the sanitation height gradient in Cambodia in these two years. Decomposition analysis, in the spirit of Blinder-Oaxaca, suggests that the reduction in children’s exposure to open defecation can statistically account for much or all of the increase in average child height between 2005 and 2010. In particular, we see evidence of externalities, indicating an important role for public policy: it is the sanitation behavior of a child’s neighbors that matters more for child height rather than the household’s sanitation behavior by itself. Moving from an area in which 100% of households defecate in the open to an area in which no households defecate in the open is associated with an average increase in height-for-age z-score of between 0.3 and 0.5. Our estimates are quantitatively robust and comparable with other estimates in the literature.

## Highlights

• Better sanitation accounts for Cambodia’s increase in child height from 2005 to 2010.
• Sanitation improvements in regions over time are associated with height improvements.
• Community open defecation matters more for child height than household open defecation.

## 1 Introduction

Child height is an important indicator of human capital and human development, in large part because of its importance for adult economic productivity and health (Currie, 2009, Vogl, 2014). This is chiefly because height is determined by health and net nutrition in the first few years of life, a critical period for cognitive development (Case and Paxson, 2008). In poor countries, the disease environment to which children are exposed and income are important indicators for adult height (Bozzoli et al., 2009). The importance for height and child development of early-life health, relative to genetics, is even greater in these countries compared to richer countries (Martorell et al., 1977, Spears, 2012b). Recent econometric evidence suggests that exposure to germs from open defecation is an important determinant of child height in developing countries (World Bank, 2008, Spears, 2013), and epidemiological evidence suggests that potential mechanisms for this relationship include diarrhea, intestinal parasites, and environmental enteropathy, a disease of the small intestine. It is therefore important to better understand the relationship among sanitation, the early-life disease environment, and subsequent child health and human capital outcomes, especially in countries where practicing open defecation is widespread.

We study the relationship between open defecation and child height in Cambodia, where 77% of households defecated in the open in 2005 and 63% in 2010. The primary contribution of this paper is to document what accounts for Cambodian children growing taller between 2005 and 2010. We show that much of the increase in child height over this period of time can be statistically accounted for by the increase in sanitation coverage over the same period.

In studying Cambodia, this paper makes three important contributions to the literature. First, in Cambodia open defecation is particularly common, representing an enduring development challenge and an unusually threatening disease environment for children. Second, although the country remains far from eliminating open defecation or child stunting, Cambodia saw an improvement in child height from 2005 to 2010, coupled with a decrease in open defecation. This improvement, which was unusually rapid among developing countries, gives us the opportunity to study any association that may exist between the two.^1^ Third, we use decomposition techniques to examine whether the change in sanitation can statistically account for the improvement in child height over this period of time.

The empirical analysis in this paper is in two parts. The main question we seek to answer is how much of the increase in child height from 2005 to 2010 can be statistically accounted for by the reduction in open defecation. We apply three complementary decomposition techniques: regression analysis to examine whether controlling for local open defecation eliminates the statistical importance of the indicator for survey year, a standard linear Blinder-Oaxaca decomposition, and a non-parametric decomposition. Although open defecation remains common in Cambodia, we find that its decline over the period studied can account for most or all of the increase in child height.

Before computing decompositions, we first explore the association between exposure to open defecation and child height over time by combining the two most recent Demographic and Health Surveys in Cambodia and using panel data methods. This analysis provides support for the association between sanitation and stunting on which we rely for computing decompositions. Using urban and rural province part fixed effects, which isolates the variation within province parts, we find that the geographic areas in which open defecation decreased by more saw a greater improvement in child height, on average. In particular, we document negative externalities of open defecation: the rate of open defecation in the child’s locality is more important for child height than the sanitation practices of the child’s own household, indicating an important role for policy as households’ own private demand for latrines may be too low.

This preliminary result is robust to a range of specifications. We test the mechanisms through which we expect open defecation to affect child height. We show that the association between open defecation and child height is steeper in urban areas, which is consistent with greater exposure to fecal pathogens when open defecation occurs in higher density areas, and that there is also an association between open defecation and child weight, consistent with mechanisms that affect child growth. We also perform two robustness checks: estimating our model by isolating the variation within regions in a particular year, and including data from the Demographic and Health Survey conducted in 2000.

### 1.1 Open defecation and child stunting

According to WHO and UNICEF Joint Monitoring Program (JMP) statistics for 2015, 13% of people in the world defecate in the open. Of these roughly one billion people, about 7.5 million live in Cambodia, representing 48% of the country’s population.^2^ Among developing countries, open defecation is particularly common in Cambodia, notably more common than in the rest of Southeast Asia, where 10% defecate in the open, sub-Saharan Africa, where 23% do, and South Asia, where 34% do.

Poor sanitation has important implications for the health and nutritional status of children, and a sizeable body of evidence in the fields of medicine and epidemiology demonstrates this link through a combination of three or more possible mechanisms, the importance of each of which may differ across contexts. These mechanisms include diarrhea, intestinal parasites, and environmental enteropathy. Checkley et al. (2004) used a cohort study in Peru to show that safe water and sanitation practices, which reduced fecal-oral contamination, were associated with fewer diarrheal episodes and better nutritional outcomes, as measured by height-for-age, in children. A meta-analysis conducted by Esrey (1996) has shown an effect of sanitation on intestinal parasites.

More recently, researchers have investigated the role of environmental enteropathy (EE) as another, and perhaps more important, mechanism linking fecal-oral contamination to malnutrition (Humphrey, 2009). EE is a largely subclinical condition that is demonstrated by damage to the walls of the small intestine thereby reducing its absorptive capacity. There is substantial evidence linking markers of EE to lower height-for-age z-scores (Kosek et al., 2013, Goto et al., 2009, Campbell et al., 2003, Lunn et al., 1991), and there is now a growing literature linking the sanitation environment to markers of EE. One recent observational study finds that indicators of EE and malnutrition are higher among children who live in “dirtier” households, where they are exposed to more fecal pathogens (Lin et al., 2013). EE may even be at play when clinical conditions like diarrhea are absent. A recent study in Mali found an effect on child height, but not on diarrhea, of a randomly assigned Community-Led Total Sanitation program (Pickering et al., 2015).

This paper joins a growing econometric literature documenting effects of open defecation on child height. Gertler et al. (2015) use experimentally-induced variation in open defecation from four randomized controlled trials (RCT) of independently conducted sanitation programs in different countries to find a causal relationship between village open defecation and child height. An RCT in Indonesia, one of the experiments studied in Gertler et al.’s meta analysis, shows that a Total Sanitation and Sanitation Marketing project increased average height of children living in households without access to sanitation at baseline (Cameron et al., 2013). Another RCT, conducted in Maharashtra, finds that improvements in sanitation brought about by the Indian government sanitation program increased average child height (Hammer and Spears, 2015). In India in particular, econometric studies of a government sanitation program document a link between sanitation and infant mortality (Spears, 2012a), child height (ibid.), cognitive achievement (Spears and Lamba, 2015), and adult wages (Lawson and Spears, 2016). In the vein of the analysis we conduct in this paper, Headey (2015) applies econometric methods to Demographic and Health Surveys in Ethiopia to identify an effect of improved sanitation on child height, and Spears (2013) investigates the difference in child height between India and Africa and documents that cross-country variation in sanitation can statistically explain a large fraction of international height differences.

Several studies of sanitation programs, however, document no health impacts. Clasen et al. (2014), for instance, find no significant health impact in an RCT studying a government sanitation program in Orissa. Patil et al. (2014) similarly find no impact in a sanitation study conducted in Madhya Pradesh. The authors of both studies note, though, that the absence of an impact on health may have been because latrine use remained low despite large increases in latrine coverage.^3^

### 1.2 Sanitation in Cambodia

Cambodia is a Southeast Asian country of 14 million people. Three-fourths of the population – and 90% of Cambodia’s poor – live in rural areas (Sobrado et al., 2014). The country has a moderate population density of about 75 people per square kilometer. Over the five-year period we study from 2005 to 2010 the fraction of the population living on less than $1.25 a day fell from around 35% to under 20%, and GDP per capita, in purchasing power parity terms, increased from $1962 to $2513 (2011 dollars), according to World Bank World Development Indicator Statistics.

Open defecation has historically been high in Cambodia. We study the change over the period from 2005 to 2010, when exposure to open defecation of the average child under five fell by about 14 percentage points from 74% of the average child’s community in 2005. Sanitation coverage also varies substantially across geographic areas within Cambodia (Robinson, 2007), another dimension of heterogeneity that this paper exploits. Unsurprisingly, given Cambodia’s high rates of open defecation relative to Southeast Asia, government, NGOs, and other partners have played an important role in development activity in Cambodia. In the period in which we study, a number of sanitation programs were active in Cambodia, mostly following a supply-driven approach to sanitation. The private sector in Cambodia has played a significant role in the provision of the majority of latrines, accounting for almost 80% of all latrines built in the country (Rosenboom et al., 2011). Although there has been some diversity of approach and method across programs and over time during this period, much of the improvement in sanitation that this paper studies reflects new latrines that were largely financed by households themselves, complemented by some subsidized provision through development programs.

## 2 Empirical strategy

Demographic and Health Surveys (DHS) are large, nationally representative surveys conducted in poor and middle-income countries. We use data on the heights of children under five years old in the two most recent DHS in Cambodia, conducted in 2005 and 2010. Our dependent variable of interest is child height-for-age, which is a z-score of a child’s distance in standard deviations from the average height of healthy children in a reference population of the same sex and age in months. We compute z-scores using the WHO’s 2006 international reference population, and follow their recommendation of omitting children beyond six standard deviations from the mean.

Our key independent variable is local area open defecation. Each household is classified as defecating in the open or not according to its report of where members “usually” defecate in the DHS questionnaire.^4^ However, infectious diseases often involve negative externalities (Gersovitz and Hammer, 2004), and intestinal disease resulting from open defecation is no exception: children are exposed to fecal pathogens from neighboring households. Therefore, we compute the fraction of households in a child’s survey Primary Sampling Unit (PSU) who defecate in the open, a continuous variable from zero to one, as a measure of “local area open defecation.”^5^ Because all households potentially contribute feces to the environment but not all households have children under five years old, we compute PSU averages from the DHS household recode. All other variables are also taken from the DHS, except measures of province mean consumption, which are from Knowles (2012), and population density, which are computed from Cambodian census data.

A concern in observational studies such as these is that sanitation improvements may have been endogenously correlated with other improvements, for instance in consumption or wealth. For this reason, we focus on conducting an exercise in statistical accounting which provides an estimate of the fraction of the change in child height that can be statistically accounted for by the simultaneous change in exposure to open defecation. We thus do not attempt to estimate a causal effect of open defecation on child height, but provide regression estimates as supporting evidence of a relationship between open defecation and child height.

In order to minimize the concern that our regression results may be driven by other factors that were simultaneously improving across Cambodia during the period of study, we employ several different strategies. First, we use geographic fixed effects. Each province is split into two parts classified as urban and rural. Urban and rural province part fixed effects, henceforth called region fixed effects, control for factors that differ across geographic areas that are correlated both with sanitation and child height. For instance, international organizations have targeted development programs in certain provinces in Cambodia. These programs may have led to sanitation improvements, but also could have improved other services or infrastructures that influence child nutrition. Region fixed effects control for such variation between regions. We also use time fixed effects to control for secular changes in child height over time. Second, it may be the case that open defecation in the child’s locality is correlated with other PSU-level variables such as village infrastructure or wealth. Thus, for comparison as placebo independent variables and as controls, we compute the fraction of households in a PSU with electricity, radio, television, refrigerator, bicycle, motorcycle, and car, as alternative measures of local living standards and infrastructure development. Third, we use a very extensive set of household controls that address maternal nutrition, household socioeconomic status, household education, and access to health care. These are discussed in more detail in Section 2.1.

### 2.1 Fixed effects identification strategy

Our identification strategy asks whether geographic areas that experienced a decrease over time in local area open defecation also experienced an increase in child height. Thus, we apply panel data methods to a pooled dataset of repeated cross sections (Deaton, 1985). The regression we estimate is:

(1) zilpt=β1local open defecationlpt+β2 household open defecationilpt           +β3mother's  heightilpt+ β4mother's  BMIilpt           +β4mother's age at  birthilpt+Bilptθ+Ppυ+PSUlptϕ+Hilptψ           +Eilptω+Vilptλ+Filptη+Milptδ+Ailpt+αp+γt+εilpt

where *i* indexes individual children, *l* is local areas (survey PSUs), *p* are 38 rural or urban parts of provinces,^6^ and *t* is time. Fixed effects α_p_ and γ_t_ are included for geographic and secular time variation.

The dependent variable, *z*, is a child’s height-for-age z-score. Local area open defecation is a fraction zero to one, and household open defecation is a binary indicator. Mother’s height, BMI, and age at birth, are included to control for heterogeneity in maternal nutrition, and to account for any possible direct effect of mother’s size (Ounsted et al., 1986). Standard errors are clustered by survey PSU, the level of heterogeneity in the independent variable of interest; 610 are more than enough for asymptotic clustered standard errors (Cameron et al., 2008).

The fixed effects identification strategy differences out any fixed heterogeneity across regions within Cambodia, as well as the secular trend in child height. We include eight further sets of control variables in stages, in order to demonstrate robustness of our regression specification and the stability of the estimates of interest:

• B_ilpt_ Birth characteristics: 13 indicators for birth order, 11 indicators for month of birth (Doblhammer and Vaupel, 2001), and whether the birth occurred in an institutional facility.
• P_pt_ Province characteristics: Province-level measures of average consumption and population density for 2005 and 2010.
• PSU_lpt_ PSU characteristics: Fraction, from zero to one, of households within the PSU with electricity, radio, television, refrigerator, bicycle, motorcycle, and car.^7^ These controls serve as both placebo independent variables and as alternative controls for PSU welfare and infrastructure.
• H_ilpt_ Household characteristics: seven binary indicators for whether the household has electricity, and owns a radio, television, refrigerator, bicycle, motorcycle, or car; ten indicators for floor material; 18 indicators for household size, ten indicators for type of cooking fuel; and 14 indicators for water source during the dry season and wet season, separately. These variables serve as additional controls for household wealth and socio-economic status.
• E_ilpt_ Education: the number of years of education completed by the father and a binary indicator for mother’s literacy.
• V_ilpt_ Vaccinations: an indicator for the child having a health and vaccination card, and eight binary indicators for the child receiving three rounds of the polio vaccine, three Diphtheria, Pertussis, and Tetanus (DPT) shots, and Bacillus Calmette–Guérin (BCG) and measles vaccines.
• F_ilpt_ Breastfeeding: a binary indicator for the child being breastfed immediately.
• M_ilpt_ Milk consumption: a binary indicator for the child being fed tinned, powdered, or fresh milk (de Beer, 2012, Baten, 2009, Baten and Blum, 2014) the previous day and/or night. This variable is only available for the youngest child under five.^8^

All specifications include 120 age-in-months times sex dummies *A_ilpt_* to non-parametrically control for the correlation between height-for-age z-score and age at measurement (Cummins, 2013).

We test the mechanisms through which we believe open defecation causes stunting using two methods. If the biological mechanisms we assume are indeed occurring, we would expect the negative impact of open defecation on child height to be greater in areas where people live nearer together and are thus more exposed to others’ fecal pathogens. We test this mechanism using our data by introducing an interaction between our open defecation variable and an indicator for whether the PSU is urban. We would also expect there to be an association between open defecation and weight-for-age if open defecation affects child nutrition by causing intestinal disease, and we test for this as well.

Finally, we perform two robustness checks. We use region by time fixed effects in order to control for time-variant regional characteristics. While this paper is primarily interested in exploring the extent to which changes in open defecation can account for changes in height over time in Cambodia, this robustness check nevertheless rules out any coincidental differences between regions over time from driving our main result. We also include data from the 2000 DHS conducted in Cambodia. Cambodia experienced a more modest decline in open defecation between 2000 and 2005 as compared to the subsequent five years. However, inclusion of data from 2000 presents an opportunity to check whether the main results of the analysis hold.

### 2.2 Decomposition of change between 2005 and 2010

If open defecation is associated with child stunting, and if open defecation became less common between 2005 and 2010, then how much of the increase in child height over this period can be statistically accounted for by the reduction in open defecation? In a separate analysis from the fixed effects estimates of the association between open defecation and child height, we approach this question with three complementary decomposition methods in Section 5. First, in the course of the regression analysis, we see that controlling for local open defecation eliminates the statistical importance of the indicator for survey year.

Second, we implement a standard linear Blinder-Oaxaca decomposition. Reimers (1983) and Jann (2008) recommend a Blinder-Oaxaca decomposition in which the estimate of the overall effect of the explanatory variable is constructed by assigning equal weight estimates to the effect computed separately from each of the two sub-samples. Then, the Blinder-Oaxaca estimate of the portion of the change in height between years that can be attributed to reduced open defecation in the child’s locality between years would be:

(2) $$\begin{array}{c} explained\;change\;in\;child\;height\;between\;2005\;and\;2010=\left(\frac{1}{2}{\hat{\beta }}_{1}^{2005}+\frac{1}{2}{\hat{\beta }}_{1}^{2010}\right)\times ({\bar{PSU\;open\;defecation}}^{2010}-{\bar{PSU\;open\;defecation}}^{2005}) \end{array}$$

Comparing this estimate to the actual change in child height between 2005 and 2010 gives the fraction accounted for by the change in open defecation.

A final, most flexible, decomposition is non-parametric reweighting (DiNardo et al., 1996).^9^ We generate a counterfactual estimate of 2005 child height by reweighting children in 2005 to match the 2010 distribution of open defecation exposure. Comparing this counterfactual estimate of the height of children in 2005 to actual child height in 2010 provides another estimate of the fraction of the difference in child height that can be accounted for by the difference in open defecation.

We construct a reweighting function, Ψ(*OD*), such that

(3) Ψ(OD)×f(OD|year=2005)=f(OD|year=2010),

where *f* is an empirical probability density function and *OD* is a vector of variables representing a child’s exposure to open defecation. Rearranging Eq. (3) gives the following:

(4) $$\Psi \left(OD\right)=\frac{f\left(OD\text{|}year=2010\right)}{f\left(OD\text{|}year=2005\right)}\text{.}$$

This function allows us to change the distribution of exposure to open defecation for 2005 children so that it matches the distribution for 2010 children.

Using this reweighting function, we may calculate the counterfactual height of children in 2005 if they had been exposed to the same levels of open defecation as children in 2010 as follows:

(5) $${\bar{height}}^{2005}=\frac{1}{n^{2010}}\overset{n^{2005}}{\underset{i}{\sum }}height_{i}\times \Psi \left(OD_{i}\right)$$

where *n^2010^* is the number of surveyed children under five in 2010*, n^2005^* is the number of surveyed children under five in 2005, and *i* indexes 2005 children.

## 3 Summary statistics

Children in our sample are on average almost two standard deviations shorter than the healthy international reference population, and they live in poor households with parents that have low levels of education. Table 1 presents sample means of many of the variables used in our analysis. Note that these summary statistics, like all estimates in this paper, are representative of children under five, and not of all Cambodians. The first and second columns show averages for 2005 and 2010, and the third column reports a test that these are different. Over the period we study, the height of children under five significantly increased relative to the international reference population while the fraction of open defecation in the average child’s community, and by individual households, significantly decreased. Standards of living also detectably improved in Cambodia between 2005 and 2010: households got richer, levels of education rose, and institutional deliveries and early initiation of breastfeeding increased.

### 3.1 Height is associated with open defecation: non-parametric descriptive regressions

**Fig. 1:**
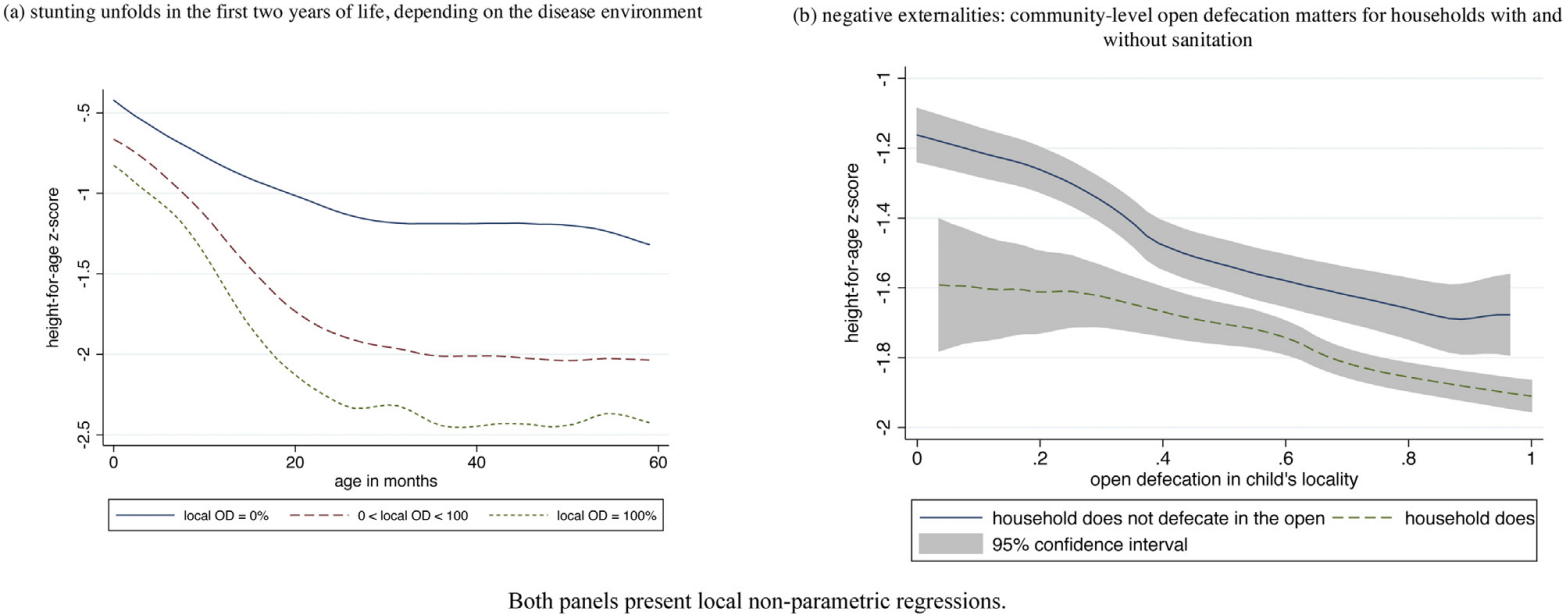
Open defecation predicts child height.

Fig. 1 presents local polynomial regressions that document that child height is associated with open defecation. Panel (a) plots child height as a function of age in months, replicating the well-known fact that most stunting occurs in the first two years of life, with height-for-age generally flat thereafter. These curves are plotted separately for children who live in local areas (PSUs) where no one surveyed defecates in the open (seven percent of children), where everyone surveyed defecates in the open (18% of children), and the rest, living in areas with an intermediate sanitation profile. Children in all three groups start off too short at birth, but the lines separate as stunting unfolds over the first two years. Children exposed to the most open defecation are more than a standard deviation shorter than children exposed to no open defecation, on average. However, open defecation is not the only cause of child growth defects: even children exposed to no open defecation are more than a standard deviation shorter than the reference population.

One reason why children exposed to better sanitation are taller is because they are also richer. They are more likely to live in households that have toilets, while poorer households are more likely to defecate in the open. However, open defecation imposes negative externalities on everyone in the vicinity, and fecal pathogens in the environment transmit disease to neighboring children. Panel (b) of Fig. 1 plots average child height-for-age of children under five as a function of local area (PSU) open defecation. Households that do and do not defecate in the open are plotted separately. Unsurprisingly, children who live in households that use toilets are taller, on average, than children who do not. However, the key feature of the graph is that both lines slant downwards. Whether or not a child’s own household defecates in the open, she is shorter, on average, if more of the households in her community do.

**Fig. 2:**
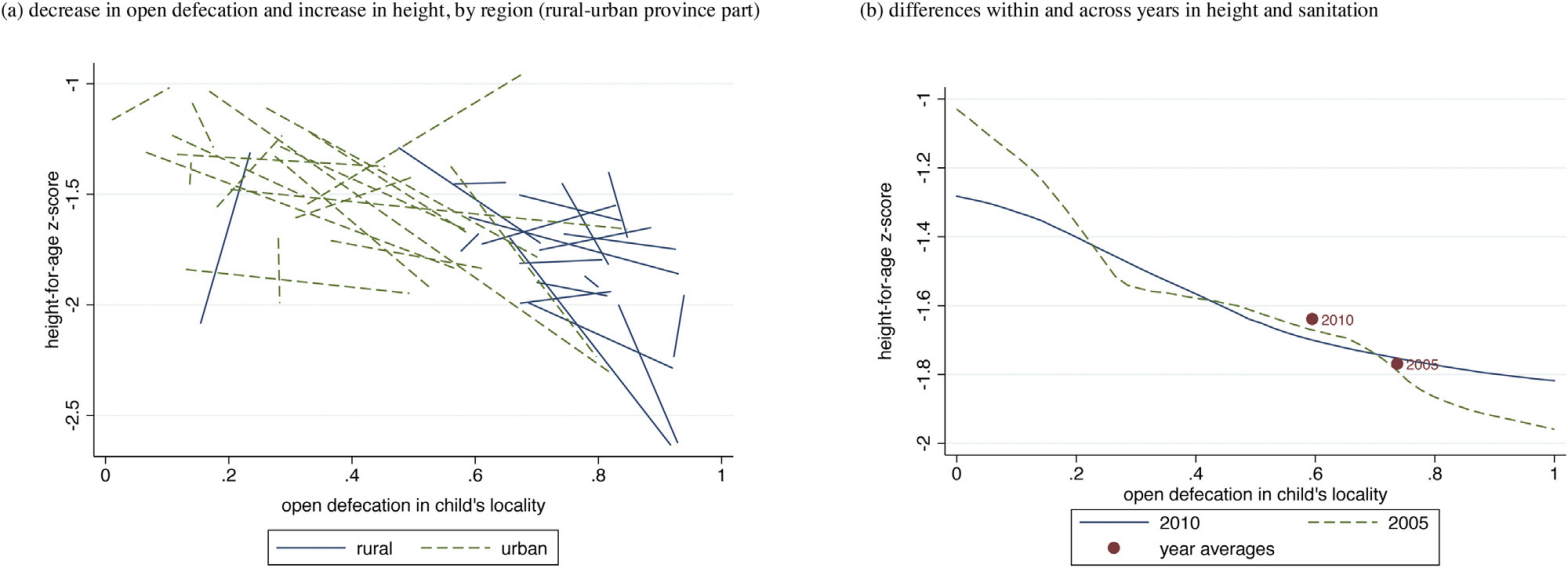
Change over time in open defecation and height, 2005–2010. In Panel (a), for each region (rural and urban part of a province), its 2005 mean for open defecation and child height is connected with its 2010 mean, to depict change over time within region. Panel (b) presents local non-parametric regressions by year, along with collapsed year averages.

What is the association between sanitation and child height over time in Cambodia? Are the geographic areas that experienced a decrease in open defecation the same areas that experienced an increase in child height? Panel (a) of Fig. 2 visually explores this question. Each line in the figure connects one region’s 2005 averages of child height and rate of open defecation in the child’s locality to its 2010 averages. Each region represents an urban or rural part of a Cambodian province. Of the 38 lines, 25 slope downwards, indicating that in 25 regions of Cambodia, average open defecation in a child’s locality reduced and average child height increased. As a preliminary non-parametric statistical significance test, a binomial distribution reports that there is only a four percent chance of seeing at least 25 of 38 lines slope downwards if these slopes are independent of one another and equally as likely to slope up or down, indicating no association at all.

The main contribution of this paper is to explore what can account for the increase in child height in Cambodia between 2005 and 2010. Panel (b) of Fig. 2 presents a visual depiction of the extent to which the change in open defecation can account for the change in child height. Within the two years 2005 and 2010, local polynomial regression lines plot average child height-for-age against open defecation rates in the locality. The lines are relatively close to one another and quite nearly pass through both of the overall year average points. Note that this is not mechanically determined: although each year’s average point must be on or near its own line, the two lines could be vertically far apart. If the lines were vertically separated, this would indicate differences in average child height for the two years even at the same level of open defecation in the locality. However, the fact that the lines are close together indicates that the association between height and sanitation in 2005 is similar to the association in 2010. Since the points are on similar lines, it appears that the within-year association between height and sanitation can statistically account for the between-years change in height. This figure is a visual representation of the results of our decomposition techniques, which we will discuss in further detail in Section 5 of this paper. Before we do so, however, we will first explore the relationship between exposure to open defecation and child height in greater detail using regression analysis.

## 4 Changes within provinces: regression evidence

This section explores whether the regions that experienced a greater decrease in open defecation also experienced a greater increase in child height between 2005 and 2010. Table 2 reports our regression results. Panel (a) shows estimates from OLS regressions without fixed effects, while Panel (b) displays results from regressions with region fixed effects. The OLS regressions identify the variation both between and within regions, while the fixed effects regressions isolate the variation occurring within regions, indicating that the relationship is not driven by coincidental differences between regions. An increase from zero to one in the rate of open defecation in the child’s locality is linearly associated with a decrease in children’s height by between 0.3 and 0.5 standard deviations, using fixed effects and varying sets of control variables.

### 4.1 Regression results

Column 1 simply reports the average improvement in child height from 2005 to 2010. Could the reduction in open defecation account for this overall increase in child height? Notably, when we introduce open defecation in the child’s locality in Column 2, the 2010 dummy variable becomes statistically and practically insignificant, indicating that the change in sanitation can statistically explain the average change in height over time.

As we progress from Column 3 to Column 6, we progressively add more control variables. Column 3 adds household open defecation, mother’s anthropometry and age at birth, birth order, month of birth, and whether the birth occurred in a facility.^10^ While measures of maternal anthropometry are unsurprisingly predictive of child height, they do not diminish the role of sanitation. In Column 4, we further add province-level consumption and density, and PSU averages for electricity coverage and radio, television, refrigerator, bicycle, motorcycle, and car ownership. Inclusion of these variables helps control for PSU-level infrastructure and wealth. Column 5 adds controls for household wealth and socio-economic status by including household-level indicators for ownership of the assets listed above, floor material, household size, type of cooking fuel, water source, father’s educational attainment, and mother’s literacy. It also includes vaccination data for polio, DPT, BCG, and measles, availability of an immunization card, and early initiation of breastfeeding. In Column 6, an indicator for milk consumption is included. This model has fewer observations because this variable is only available for the youngest child under five, rather than for all children under five. For this reason, Column 5 represents the authors’ preferred specification. All specifications include 120 age-in-month dummies, separately for boys and girls.^11^^,^^12^^,^^13^^,^^14^

Three conclusions emerge from the table. The first is the robustness of the coefficient on open defecation in the child’s locality. The variable remains highly statistically significant in all specifications. If instead of clustering standard errors at the PSU level, we cluster at the more conservative level of 38 regions, the *t*-statistic on open defecation in the child’s locality in the most controlled specification, Column 5 of Panel (b), becomes −2.75, although this may be too few clusters for asymptotic results (Cameron et al., 2008).

Secondly, the clear similarity in the size of coefficients from the OLS and fixed effects regressions suggests that the results from the OLS regressions are not a spurious artifact of heterogeneity across regions. Fixed effects are well-known to risk attenuation bias. However, that appears to be unlikely in this case due to the small differences between corresponding models of Panel (a) and Panel (b).^15^

Finally, an important result for policy is that the coefficient on *household* open defecation is not statistically significant.^16^ This is unsurprising because open defecation, like other sources of infectious disease, involves important negative externalities. In Table 3, we split the sample by whether or not the household in which the child lives defecates in the open. Open defecation in the community predicts child height, regardless of whether the household defecates in the open. This result corroborates the importance of negative externalities (Geruso and Spears, 2015). Because such externalities are a classic economic rationale for public action, they point to the importance of a policy response to open defecation.

### 4.2 Mechanism check: steeper slope in urban areas

The importance of open defecation in the child’s locality, rather than the household’s own open defecation, indicates a key role for externalities of disease. If so, then because children are more exposed to others’ fecal pathogens where people live nearer together, we would further expect that open defecation should have a steeper association with child height in urban areas, where population density is particularly high (Hathi et al., 2014, Bateman et al., 1993, Bateman and Smith, 1991). We test this by introducing an interaction between prevalence of open defecation in the locality and an urban dummy to the specification in Column 2 of Table 2.

Does open defecation indeed have a steeper association with child height in urban parts of Cambodia than in rural parts? The fraction of open defecation in the PSU interacts with an indicator for urban place, with and without fixed effects. An increase in the rate of open defecation in the child’s locality from zero to one is associated with a 0.34 (s.e. 0.16) standard deviation greater decrease in average child height in urban areas compared to rural areas using a standard OLS model, and a 0.41 (s.e. 0.22) standard deviations greater decrease using a model with region fixed effects. These coefficients are statistically significant at the two-sided ten percent level. This finding is consistent with Spears (2012a), Spears (2013), and Hathi et al. (2014).

### 4.3 Mechanism check: weight-for-age

If open defecation does indeed affect child height by causing intestinal diseases that lead to undernutrition, then we may simultaneously expect to see an effect of open defecation on weight, another measure of nutritional status. Weight-for-age is associated with recent diarrheal episodes (Schmidt et al., 2010) and environmental enteropathy (Lin et al., 2013, Humphrey, 2009). As a measure of nutrition, weight-for-age is more responsive to recent changes in diet, care practices, or the disease environment, while height-for-age is a measure of *net* nutrition in the first two years of life (Waterlow, 1972, Black et al., 2008).

Open defecation in the child’s locality statistically significantly predicts weight-for-age, with and without fixed effects. Replacing height-for-age with weight-for-age in Column 3 of Table 2 shows that an increase in open defecation in the child’s locality from zero to one is associated with a reduction in weight of 0.56 (s.e. 0.083) standard deviations in a standard OLS model, and 0.23 (s.e. 0.074) standard deviations in a model with region fixed effects. These coefficients are significant at the two-sided one percent level.

### 4.4 Robustness checks

We test the robustness of our results using two methods: including region by time fixed effects in order to isolate the variation occurring within regions in a particular year, and adding data from the DHS conducted in Cambodia in 2000. Table 4 reports the results of our robustness checks. In Column 1, we include region by time fixed effects. This more restrictive specification changes the coefficient on open defecation in the child’s locality only very slightly: using the same set of controls as in Column 5 in Table 2, the associated reduction in child height arising from an increase in open defecation in the child’s locality from zero to one is 0.35 standard deviations using region fixed effects (Table 4, Column 1) and 0.29 using region by time fixed effects (Table 4, Column 2).

Column 3 of Table 4 includes data from the 2000 Cambodian DHS. Between 2000 and 2005, Cambodia experienced a much more modest decline in open defecation of only eight percentage points, compared to the subsequent five years in which the decline was 14 percentage points. Nevertheless, including data from the year 2000 only supports our main result.

## 5 Decomposition of the 2005–2010 increase in child height

How much of the increase in child height between 2005 and 2010 can be explained by the decrease in average exposure to open defecation? Econometric decompositions ask how much of the difference in the outcome variable across two groups can be accounted for by observable differences in input variables (Fortin et al., 2011). Although the canonical use of decompositions in labor economics is to analyze differences in economic outcomes (such as wages) between two groups (such as black and white people in the United States), here we will be asking how much of the difference in child height in Cambodia between 2005 and 2010 can be accounted for by the difference in the level of open defecation in a child’s locality. In general, econometric decompositions of observational data are tools of statistical accounting that may or may not have a causal interpretation depending on the details of the data and the source of heterogeneity studied. We thus interpret decomposition results conservatively as accounting for differences.

In Section 3.1, we discussed Panel (b) of Fig. 2, which presents a visual depiction of the extent to which the change in open defecation can explain the change in child height. Each line plots local polynomial regressions of the sanitation height gradient for each year. The relative closeness of the lines indicates that the gradient is similar in both years, and the fact that the overall year averages for both years are almost on these lines indicates that the within-year association between height and sanitation appears to statistically account for the between-years change in height.

Various methods of econometric decomposition are available, and we study three. The first and simplest was already presented in the difference between Columns 1 and 2 of Table 2. Adding a linear control for open defecation in a child’s locality (PSU mean open defecation) eliminates a statistically significant difference in child height between the two DHS rounds. Using OLS and fixed effects estimation strategies, controlling for open defecation in the child’s locality statistically accounts for 88% and 86% of the difference in child height from 2005 to 2010, respectively (see Table 5). The following two sub-sections will consider a Blinder (1973) – Oaxaca (1973) decomposition, and will apply a non-parametric reweighting technique.

### 5.1 Blinder-Oaxaca decomposition

Blinder-Oaxaca decomposition calculates the explained change in child height between 2005 and 2010 using an estimate of the height-sanitation gradient. In this case, two regressions separately estimate β_1_^2005^ and β_1_^2010^ and then average them to create a counterfactual within-year height sanitation slope. Multiplying this by the difference in the average exposure to open defecation between 2005 and 2010 provides an estimate of the change in child height that can be accounted for by the change in open defecation.

As Table 5 shows, this approach finds that the reduction in open defecation to which the average child was exposed can statistically account for 0.12 of the 0.13 standard deviation difference in child height. Thus, 92% of the difference in height can be accounted for by the difference in sanitation.

### 5.2 Non-parametric reweighting decomposition

This method creates a counterfactual average height for children in 2005, reweighted to match the 2010 distribution of open defecation. In particular, the sample is split into 12 bins of PSU open defecation levels: ten deciles with extra categories for open defecation of zero and open defecation of one. These are crossed with an indicator for own household open defecation to create 22 overall open defecation bins (there are 22 instead of 24 because there are no children in households that openly defecate who also live in PSUs where nobody sampled defecates in the open, and vice versa). Then, within each of the 22 bins, the total sample weight is computed separately for 2010 and 2005. A new set of weights is computed for children in 2005 by multiplying their sampling weight by the ratio of their bin’s 2010 total sampling weight to their bin’s 2005 total sampling weight. Finally, the new weighted average represents the counterfactual height of children in 2005 if they had been exposed to the same levels of open defecation as children in 2010.

This result is compared with the other decomposition methods in Table 5. The true sample mean height for age was −1.77 in 2005 and −1.64 in 2010. When the 2005 sample is reweighted to match the 2010 sanitation distribution, the counterfactual mean height-for-age is −1.63, essentially the same as the true 2010 average (the difference is not statistically significant, *t* = 0.29).

Therefore, all three approaches to decomposing the change over time in child height reach similar conclusions. Using simple pooled regression, a Blinder-Oaxaca decomposition, or non-parametric reweighting, the decline in exposure to open defecation can statistically account for almost all of the approximately 0.13 standard deviation increase in height-for-age.

## 6 Conclusion

Child height is an important economic variable predicting adult human capital, cognitive achievement, and health. The average child under five in Cambodia was 0.13 standard deviations taller in 2010 than in 2005. Decomposition analysis finds that much of the increase in child height between 2005 and 2010 can be accounted for by the simultaneous reduction in open defecation. At the same time, regression analysis finds a robust and large association between exposure to open defecation and child height. The point estimates computed in this analysis are consistent with other studies. A meta-analysis combining data from three large-scale randomized interventions conducted independently in India, Indonesia, and Mali finds that eliminating open defecation in a village in which everyone practices open defecation is associated with a 0.4 standard deviation increase in height (Gertler et al., 2015). This is similar to the point estimates found in this paper of between 0.3 and 0.5 standard deviations, with region fixed effects and controls.

The change in child height over this period of time represents an important difference: Spears’ (2012b) estimates of the height-cognitive achievement gradient for Indian children suggest that a 0.13 standard deviation increase in child height would be associated with a 1–4 percentage point increase in the probability of being able to read words or paragraphs among 8–11 year-olds. This difference is also quantitatively similar to the India-Africa height gap (Spears, 2013).

These results indicate that widespread open defecation could be a critical constraint for human development. Moreover, we have seen various indicators of the role of negative externalities in propagating fecal pathogens. The health benefits of better sanitation are significant, and in Cambodia, the cost associated with constructing a latrine can be as low as $25 (Rosenboom et al., 2011). Lawson and Spears (2016) find a robust relationship between adult wages and the disease environment during childhood in India, and the fiscal implications indicate that public investment in sanitation infrastructure may come at very low net present cost. Interventions that are long-term are more likely to lead to sustainable improvements in nutritional indicators for children (Reiger and Wagner, 2015). Thus, if latrine adoption is durable, it can have a substantial impact on child height.

Between 2005 and 2010, open defecation decreased, and child height increased, but open defecation is still common in Cambodia and the mean child was still 1.64 standard deviations below the healthy reference population in 2010. In any country where this is the case, spillovers of poor sanitation indicate that reducing open defecation must be a policy priority.

## Figures and Tables

**Table 1:** Summary statistics: Improvements in height, sanitation, and living standards.

	2005	2010	t-statistic 2005 = 2010
height-for-age z-score	−1.77	−1.64	2.66
	0.0355	0.0335	
open defecation in child’s locality	0.737	0.595	−6.56
	0.0158	0.0163	
household open defecation	0.772	0.627	−6.03
	0.0164	0.0184	
mom’s height	152.6	152.8	0.91
	0.130	0.144	
mom’s BMI	20.8	21.0	1.39
	0.0716	0.0722	
mom’s age at birth	27.5	26.5	−4.21
	0.172	0.152	
non-institutional delivery	0.781	0.464	−14.19
	0.0148	0.0169	
urban locality	0.140	0.157	0.84
	0.0167	0.0165	
consumption	3756	5148	12.51
	81.2	77.9	
density	143	167	1.34
	11.3	13.9	
household electrification	0.180	0.287	4.15
	0.0173	0.0207	
household radio ownership	0.437	0.384	−2.70
	0.0144	0.0136	
household TV ownership	0.494	0.581	3.75
	0.0170	0.0168	
household refrigerator ownership	0.0196	0.0466	3.38
	0.00453	0.00699	
household bicycle ownership	0.6197	0.5874	−1.51
	0.0141	0.0156	
household motorcycle ownership	0.350	0.555	9.30
	0.0160	0.0152	
household car ownership	0.0371	0.0637	2.92
	0.0056	0.0075	
mom’s literacy	0.463	0.457	−0.27
	0.0157	0.0161	
father’s years of education	5.11	5.80	3.61
	0.142	0.136	
breastfeeding initiated immediately	0.345	0.613	12.27
	0.0148	0.0153	
n (children under 5)	3587	3699	

Table presents sample means. Standard errors clustered by survey primary sampling unit below.

**Table 2:** Regression Results: Open defecation in the child’s locality predicts child height.

	(1)	(2)	(3)	(4)	(5)	(6)
(a): Without region (rural and urban province part) fixed effects						
open defecation in child’s		−0.814***	−0.559***	−0.254**	−0.294**	−0.358**
locality		(0.0656)	(0.0832)	(0.109)	(0.120)	(0.154)
year 2010	0.132***	0.0162	−0.0165	0.0477	0.0422	0.0482
	(0.0445)	(0.0444)	(0.0431)	(0.0494)	(0.0559)	(0.0718)
household open defecation			−0.0255	−0.0232	0.0652	0.124
			(0.0561)	(0.0540)	(0.0574)	(0.0755)
mom’s height			0.0588***	0.0570***	0.0539***	0.0549***
			(0.00332)	(0.00332)	(0.00358)	(0.00441)
mom’s BMI			0.0291***	0.0285***	0.0234***	0.0210**
			(0.00643)	(0.00642)	(0.00669)	(0.00873)
mom’s age at birth			0.0119***	0.00975**	0.00824*	0.00615
			(0.00415)	(0.00418)	(0.00442)	(0.00547)
urban				−0.0837	−0.0975	−0.195**
				(0.0586)	(0.0619)	(0.0798)
birth characteristics			yes	yes	yes	yes
province characteristics				yes	yes	yes
PSU characteristics				yes	yes	yes
household characteristics					yes	yes
parents' education					yes	yes
vaccination					yes	yes
breastfeeding					yes	yes
milk consumption						yes
n (children under 5)	7286	7286	7168	7168	6389	4213

(b): With region (rural and urban province part) fixed effects						
open defecation in child’s		−0.738***	−0.502***	−0.289***	−0.350***	−0.386**
locality		(0.0824)	(0.0927)	(0.111)	(0.120)	(0.156)
year 2010	0.123***	0.0167	−0.0153	0.0891	0.0865	0.00740
	(0.0430)	(0.0420)	(0.0415)	(0.143)	(0.152)	(0.199)
household open defecation			−0.0180	−0.0125	0.0816	0.136*
			(0.0559)	(0.0539)	(0.0572)	(0.0755)
mom’s height			0.0566***	0.0557***	0.0530***	0.0531***
			(0.00331)	(0.00331)	(0.00358)	(0.00434)
mom’s BMI			0.0314***	0.0305***	0.0259***	0.0241***
			(0.00653)	(0.00647)	(0.00677)	(0.00883)
mom’s age at birth			0.0115***	0.0102**	0.00830*	0.00568
			(0.00410)	(0.00416)	(0.00445)	(0.00559)
birth characteristics			yes	yes	yes	yes
province characteristics				yes	yes	yes
PSU characteristics				yes	yes	yes
household characteristics					yes	yes
parents' education					yes	yes
vaccination					yes	yes
breastfeeding					yes	yes
milk consumption						yes
n (children under 5)	7286	7286	7168	7168	6389	4213

Standard errors clustered by survey primary sampling unit in parentheses. Two-sided *p*-values: *** p < 0.01, ** p < 0.05, * p < 0.1. The dependent variable in all regressions is height-for-age z-score. All specifications include 120 age-in-month dummies, separately for boys and girls. Birth characteristics include birth order, month of birth, and whether delivery occurred in an institution. Province characteristics include consumption and density at the province level. PSU characteristics include average electricity coverage and average ownership of radio, television, refrigerator, bicycle, motorcycle, and car. Household characteristics include household electricity, ownership of the same set of assets, floor material, cooking fuel, water source during dry and wet seasons, and number of household members. Parents’ education includes mother’s literacy and father’s number of years of education. Vaccination includes whether the child has an immunization card, and binary indicators for having received vaccinations for BCG, DPT, polio, and measles. Breastfeeding indicates that breastfeeding was initiated immediately. Milk consumption indicates that the child was given tinned, powdered, or fresh milk the previous day and/or night.

**Table 3:** Externalities: community-level open defecation matters for households with and without toilets.

	*(1)*^a^	(2)	(3)
specification same as	Table 2, Panel (b), Column 5		
sample includes children in households	*all*	without toilets	with toilets
open defecation in child’s locality	−0.350***	−0.348*	−0.557***
	*(0.120)*	(0.182)	(0.201)

n (children under 5)	*6389*	4425	1964

Standard errors clustered by survey primary sampling unit in parentheses. Two-sided *p*-values: *** p < 0.01, ** p < 0.05, * p < 0.1. All specifications include 120 age-in-month dummies, separately for boys and girls.

a This is the same model as Table 2, Panel (b), Column 5. Coefficient is repeated for comparison purposes.

**Table 4:** Robustness: The importance of open defecation is robust to different model specifications.

	*(1)*^a^	(2)	(3)
control variables same as in Table 2,	*Column 5*	Column 5	Column 5^b^
open defecation in child’s locality	−0.350***	−0.292**	−0.335***
	*(0.120)*	(0.117)	(0.126)

region by time FE		**yes**	
region FE	*yes*		yes
2000 data			**yes**
2005 data	*yes*	yes	yes
2010 data	*yes*	yes	yes
n (children under 5)	*6389*	6389	9698

Standard errors clustered by survey primary sampling unit in parentheses. Two-sided *p*-values: *** p < 0.01, ** p < 0.05, * p < 0.1. All specifications include 120 age-in-month dummies, separately for boys and girls. In each column, the difference from Table 2, Column 5 are in bold.

a This is the same model as Table 2, Panel (b), Column 5. Coefficient is repeated for comparison purposes.

b Province-part characteristics (consumption and density) have been excluded from this model as they are not available for the year 2000. Water source has also been excluded because of inconsistencies in data collection from 2005 to 2010.

**Table 5:** Decomposition results: Fraction of 2005–2010 height change explained by improving sanitation.

	difference between 2005 and 2010 mean heights		
decomposition method	before sanitation	after sanitation	percent explained
regression, OLS	0.132	0.0162	88%
regression, fixed effects	0.123	0.0167	86%
Blinder-Oaxaca	0.129	0.0107	92%
reweighted mean	0.129	−0.0121	109%

Regression results are reinterpreted from Table 2. “Before sanitation” is the simple average difference; “after sanitation” is the unexplained difference after accounting for the improvement in open defecation. “Blinder-Oaxaca” is a two-way decomposition with equal weight on within-sample slopes. “Reweighted Mean” constructs a counterfactual mean 2005 height by reweighting the 2005 sample to match the sanitation distribution of the 2010 sample; the after difference is negative because the counterfactual 2005 height is slightly greater than the real 2010 height.

## Footnotes

1 In Cambodia, open defecation rates fell by 14 percentage points over the five-year period studied. According to WHO-UNICEF Joint Monitoring Program data, Cambodia’s decline in open defecation over this period was in the 96th percentile of all countries in the world.

2 The JMP compiles data from multiple sources including DHS and country censuses, and makes projections for years in between using a linear trend. For this reason, estimates from the JMP often differ from DHS data, on which this paper relies.

3 The authors of the Orissa study write: “insufficient coverage and use of latrines seem to be the most likely causes for the absence of effect” (Clasen et al., 2014). Similarly, the authors of the Madhya Pradesh study state: “the less than universal or very high levels of IHL coverage in the intervention villages combined with relatively small behavior changes are consistent with our finding of no improvements in child health outcomes” (Patil et al., 2014).

4 Since the DHS collect defecation behavior at the household level, we are unable to construct a measure of local area open defecation based on individual behavior. In India, Coffey et al. (2014) and Coffey et al. (2015) find substantial variation in latrine use even among individuals living in households with latrines. To our knowledge, no similar specialized survey data exist for Cambodia.

5 The Cambodian DHS uses Census enumeration areas (EAs) as its sampling frame. EAs are composed of an entire urban or rural “village” or of a part of a large urban or rural “village.” In 2010, the average number of households per enumeration area was 98 households. Given that population density was 75 persons per square kilometer and average household size was 4.7, as measured in the 2008 General Population Census, we estimate that the average village was 6.1 km^2^. In 2005, the average number of households per EA was less than 161, population density was 74 persons per square kilometer, and average household size was 5.1. Thus, the average village was around 11.1 km^2^. This computation of local area open defecation may not perfectly reflect a child’s exposure to open defecation; there may be measurement error. This measurement error is expected to be random because the DHS employs a simple random sampling procedure to select households within PSUs. Random measurement error in an independent variable only attenuates results. Attenuation is further discussed in Section 4.1.

6 Thus, provinces with both rural and urban data in the DHS have two index numbers within *p* and these parts each have their own separate fixed effect; provinces with only rural or only urban households have one fixed effect.

7 Between 14 and 28 households were surveyed in each PSU. In both 2005 and 2010, 25.6 households on average were surveyed per PSU.

8 The DHS does not have a consumption module and detailed information about calorie consumption is not available. Additionally, infant and young child feeding indicators are not directly comparable across Cambodia’s 2005 and 2010 survey rounds. However, according to the official report for the 2010 Cambodian DHS, this is unlikely to threaten any bias for our results, because “There has not been much improvement in complementary feeding practices since 2005” (ICF Macro, 2011).

9 Coffey (2015) uses this method to estimate the fraction underweight of pre-pregnant women in India, Spears (2013) utilizes it to estimate the fraction of the India-Africa average height gap that is attributable to open defecation, and Geruso (2012) employs it to estimate the extent to which socio-economic status can account for the difference in life expectancy between blacks and whites in the United States.

10 Guiteras et al. (2015) find that household open defecation depends on open defecation in the community in Bangladesh, raising concerns over multicollinearity. However, the stability of the coefficient on local area open defecation across models mitigates this concern.

11 The DHS does not include measures of calorie consumption, but overall household food availability would likely be at least partially reflected in mother’s BMI, controlling for which does not change our result.

12 We also tested for a nonlinear relation between open defecation and child height by introducing a quadratic term for open defecation in the child’s locality. The coefficient on the quadratic term was not statistically significant, and the addition of this term did not improve the fit of the model.

13 We also tested for whether the relationship between open defecation and child height was different between boys and girls, and whether there was an interaction between local area open defecation and household open defecation. Neither appear to be significant.

14 As suggested by Fig. 1, open defecation is more steeply associated with child height at older ages; we find a statistically significant interaction between age and local area open defecation. This interaction supports a similar finding by Spears (2013) in India.

15 Nevertheless, attenuation bias may also result from measurement error in independent variables. Local open defecation rates contain measurement error because they are computed sample means and may not perfectly reflect the true exposure to fecal bacteria experienced by children (Deaton, 1985). To respond to this concern, we instrument for the same PSU-level open defecation independent variable with another measure of the local disease environment: *district*-level open defecation. The time and place fixed effects estimate in Column 2 of Panel (b) rises in absolute value from −0.74 without instrumenting to −1.02 with instrumenting (s.e. = 0.37), or an estimate of −1.18 with instrumenting and all of the controls in Column 5 of Panel (b). Thus, if attenuation bias is indeed a problem here, the true effect of open defecation on child height may be greater than we have estimated.

16 This finding is also econometrically important as a reminder that statistical approaches which study the “effects” of a child’s own household’s open defecation are likely to both importantly overlook these external effects and conflate heterogeneity in wealth. For example, a recent analysis of child height in Cambodia by Ikeda et al. (2013) includes an indicator for household sanitation as an independent variable, but they omit any measure of local or otherwise geographic sanitation. Unsurprisingly, they find, as we do, that household sanitation is not a particularly important predictor of child height after including other measures of household wealth.
